# Overexpression of lncRNA IGFBP4–1 reprograms energy metabolism to promote lung cancer progression

**DOI:** 10.1186/s12943-017-0722-8

**Published:** 2017-09-25

**Authors:** Binyao Yang, Lisha Zhang, Yi Cao, Shuai Chen, Jun Cao, Di Wu, Jiansong Chen, Huali Xiong, Zihua Pan, Fuman Qiu, Jinbin Chen, Xiaoxuan Ling, Maosheng Yan, Suli Huang, Shiyu Zhou, Tiegang Li, Lei Yang, Yunchao Huang, Jiachun Lu

**Affiliations:** 1 The State Key Lab of Respiratory Disease, The institute for Chemical Carcinogenesis, Collaborative Innovation Center for Environmental Toxicity, Guangzhou Medical University, 195 Dongfengxi Road, Guangzhou, 510182 China; 2 Department of Central Laboratory, The 5th Affiliated Hospital of Guanzhou Medical University, Guangzhou, 510700 China; 3 Yunnan Province Tumor Hospital, the Third Affiliated Hospital of Kunming Medical University, Kunming, 650118 China; 4 The First People’s Hospital of Qujing, Qujing, 655000 China; 5 Guangdong Province Hospital for Occupational Disease Prevention and Treatment, 68 Haikang Road, Guangzhou, 510300 China; 6 Shenzhen Center for Disease Control and Prevention, Shenzhen, 518055 China

**Keywords:** lncRNA, Lnc-IGFBP4–1, Proliferation, Metastasis, Energy metabolism, Lung cancer

## Abstract

**Background:**

Reprogrammed energy metabolism as an emerging hallmark of cancer has recently drawn special attention since it facilitate cell growth and proliferation. Recently, long noncoding RNAs (lncRNAs) have been served as key regulators implicated in tumor development and progression by promoting proliferation, invasion and metastasis. However, the associations of lncRNAs with cellular energy metabolism in lung cancer (LC) need to be clarified.

**Methods:**

Here, we conducted bioinformatics analysis and found insulin-like growth factor binding protein 4–1 (IGFBP4–1) as a new candidate lncRNA located in the upstream region of IGFBP4 gene. The expression levels of lnc-IGFBP4–1, mRNA levels of IGFBP4 in 159 paired lung cancer samples and adjacent, histological normal tissues by qRT-PCR. Over-expression and RNA interference (RNAi) approaches were adopted to investigate the biological functions of lnc-IGFBP4–1. The intracellular ATP level was measured using the Cell Titer-Glo Luminescent Cell Viability Assay kit, and changes in metabolic enzymes were examined in cancer cells and normal pulmonary epithelial cells with qRT-PCR.

**Results:**

Our results showed that lnc-IGFBP4–1 was significantly up-regulated in LC tissues compared with corresponding non-tumor tissues (*P* < 0.01), and its expression level was significantly correlated with TNM stage (*P* < 0.01) and lymph node metastasis (*P* < 0.05). Further investigation showed that overexpression of lnc-IGFBP4–1 significantly promoted LC cell proliferation in vitro and in vivo, while downregulation of endogenous lnc-IGFBP4–1 could inhibited cell proliferation and induce apoptosis. Moreover, we found lnc-IGFBP4–1 could influences ATP production levels and expression of enzymes including HK2, PDK1 and LDHA, in addition, decline in both ATP production and these enzymes in response to 2-DG and 2-DG-combined Rho123, respectively, was observed in lnc-IGFBP4–1-overespressing LC cells, indicative of an enhanced aerobic glycolysis rate. Finally, lnc-IGFBP4–1 was observed to negatively correlate with gene IGFBP4, and lower expression level of IGFPB4 was found after lnc-IGFBP4–1-overexpression was transfected into PC9 cells, higher expression level of IGFPB4 was also found after lnc-IGFBP4–1-downregulation was transfected into GLC-82 cells, which indicates that IGFBP4 may exert its targeting function regulated by lnc-IGFBP4–1.

**Conclusions:**

Taken together, these findings provide the first evidence that lnc-IGFBP4–1 is significantly up-regulated in LC tissues and plays a positive role in cell proliferation and metastasis through possible mechanism of reprogramming tumor cell energy metabolism, which suggests that lnc-IGFBP4–1 may be a promising biomarker in LC development and progression and as a potential therapeutic target for LC intervention.

**Electronic supplementary material:**

The online version of this article (10.1186/s12943-017-0722-8) contains supplementary material, which is available to authorized users.

## Background

Lung cancer (LC) is the most common cause of global cancer-related death with an approximate 5-year survival rate of 16.6% all over the world [1]. Recently, although great advance has been made in clinical treatment for LC, the overall survival time of LC patients has not improved dramatically and a critical problem for that is the lack of valuable molecular biomarkers. Thus, a good understanding of the molecular mechanism underlying LC progression and metastasis is urgent to improve the diagnosis and effective therapy at the onset of the disease. Long non-coding RNAs (lncRNAs) as one of the non-protein coding transcripts are commonly defined as longer than 200 nucleotides in length [2] and have recently attracted increasing attention. LncRNAs served as important players have been implicated in a variety of physiological and pathological processes, and the majority, so far, studied are implicated in gene regulation either at the transcriptional or posttranscriptional level [3]. In cancer, abnormal expression and mutations of lncRNAs as important regulators can contribute to tumor development and progression through pathophysiological activities such as cell growth, apoptosis, invasion, and metastasis [4–8]. As an emerging hot spot of cancer research, numerous cancer-specific lncRNAs have been identified, among which several have been validated as biomarkers for metastasis or metabolism, such as metastasis associated long antisense transcript 1 (MALAT-1) [9], prostate cancer gene expression marker 1 (PCGEM1) [10] and HOX transcript antisense RNA (HOTAIR) [11]. Here, we found an lncRNA termed insulin-like growth factor binding protein 4–1 (IGFBP4–1) located in the upstream region of IGFBP4 gene by bioinformatics analysis. IGFBPs is a kind of multifunctional cell proliferation regulation factor, which play an important role in tumor metabolic processes via competing with insulin-like growth factor receptor (IGFR) to combine with insulin-like growth factors (IGFs) and regulate the biological function of the IGFs eventually [12, 13]. IGFBP4 is an important core member of the IGFBPs family, which can mediate its main functions through inhibiting IGF-induced cellular growth and thus regulate the tumor metabolic processes [14]. Several studies have found that overexpression of IGFBP4 inhibited the cell growth in some cancers including prostatic cancer [15], colon cancer [16] and breast cancer [17]. It has been found that higher amounts of IGFBP4 mRNA in normal lung than in tumor tissues derived from lung cancer patients, and the expression level of IGFBP4 is in association with tumor differentiation, the poorly differentiated adenocarcinoma cells often lost their IGFBP-4 expression [18–20]. Given this, recent research therefore planned to emphasize mechanisms mediated by this lncRNA in cancer progression.

Conceptual progress has made us to better understand that the chronic and uncontrolled cell proliferation and metastasis are representative of the essence of LC involves not only deregulated control of cell proliferation but also making corresponding adjustments of energy metabolism in order to accelerate cell growth and division. Reprogrammed energetic metabolism as a result of increased glycolysis and glucose uptake to facilitate cell growth and proliferation was recently pointed out as an emerging hallmark of cancer [21]. Most cancer cells employ aerobic glycolysis coupling with reduced mitochondrial oxidative phosphorylation for energy instead of oxidative phosphorylation, even in sufficient oxygen state. This phenomenon is called “Warburg effect”, increasing uptake of glucose and glutamine; synthesis of more ATP, amino acids, nucleic acids, and lipids; and lead to changes in the activity of relevant enzymes in the process of glucose metabolism [22]. To compensate for the consequent decrease in ATP production, cancer cells adopt corresponding mechanisms to increase glucose uptake and utilization. One mechanism underlying the regulation of glucose transporters, especially GLUT1, is responsible for increase glucose uptake in the cytoplasm [23, 24]. Here we focus on a highly possible mechanism mediated by lncRNA IGFBP4–1 to promote proliferation and metastasis through reprogramming glucose metabolism in LC.

Evidence from other studies demonstrates that lncRNA is involved in tumor metabolic regulation process. LncRNA PCGEM1 functioned as a unique target to regulate energy metabolism for prostate-cancer therapy [10]. Lnc-UCA1 plays a positive role in cancer cell glucose metabolism through the cascade of mTOR-STAT3/microRNA143-HK2, and reveal a novel link between lncRNA and the altered glucose metabolism in cancer cells [25]. Given that lncRNA may be functioned as an important regulator of cancer energy metabolism that promotes to improve biosynthetic processes, supporting proliferative advantages for rapid cancer cell growth. Thus, more comprehensive assessment of the impact of lnc-IGFBP4–1 on the metabolic features of LC cells may shed new light on the molecular mechanism how lnc-RNA exerts influence to regulate metabolic programming to facilitate the cancer cell growth and metastasis. In the present study, we observed that lnc-IGFBP4–1 is up-regulated in LC tissues compared with corresponding non-tumor tissues and that its expression level is significantly correlated with TNM stage and lymph node metastasis. Subsequently, we found lnc-IGFBP4–1 could regulate cell growth and metastasis both in vitro and in vivo. In addition, we found that lnc-IGFBP4–1 negatively correlated to the gene IGFPB4 and IGFBP4 expressiong levels was decreased in lnc-IGFBP4–1-overexpressing cells. Moreover, lnc-IGFBP4–1 was determined to be as an important regulator in the process of ATP production and enzymatic activities in LC.

## Methods

### Subjects and tissue samples

The 159 lung cancer tissues and corresponding adjacent normal lung tissues were collected from patients of the First Affiliated Hospital of Guangzhou Medical University, the second Affiliated Hospital of Guangzhou Medical University, and the Third Affiliated Hospital of Kunming Medical University from May 2013 to December 2015. The clinical characteristics were presented in Table 1. The samples were snap-frozen in liquid nitrogen immediately after resection. Detailed information about demography, clinical characteristics and histopathology was collected for all patients. The diagnosis of lung cancer was confirmed by histopathology. All patients were staged based on the International Association for the Study of Lung Cancer (IASLC) Tumor-Node-Metastasis (TNM) classification. All the patients involved have no genetic connections. This study was reviewed and approved by the Ethics Committee of Guangzhou Medical University, and all patients provided written informed consent.

**Table 1:** Correlation between lncRNA *IGFBP4-1* expression and clinicopathological characteristics of LC patients

Characteristics	N of cases	Relative IGFBP4–1 expression		
		Low	High	*P*-value^a^
Age(years)				0.482
≤ 60	96	17 (54.8)	79 (61.7)	
> 60	63	14 (45.2)	49 (38.3)	
Gender				0.201
male	61	15 (48.4)	46 (35.9)	
female	98	16 (51.6)	82 (64.1)	
Smoking History				0.304
smokers	69	16 (51.6)	53 (41.4)	
never smokers	90	15 (48.4)	75 (58.6)	
Drinking History				0.156
drinkers	95	22 (71.0)	73 (57.0)	
never drinkers	64	9 (29.0)	55 (43.0)	
TMN stage				0.007*
I+ II	89	24 (77.4)	65 (56.0)	
III + IV	70	7 (22.6)	63 (44.0)	
T status				0.041*
T1 + T2	109	26 (83.9)	83 (64.9)	
T3 + T4	50	5 (16.1)	45 (35.1)	
N status				0.044*
negative (N0))	62	17 (54.8)	45 (35.2)	
positive (N1 + N2 + N3)	97	14 (45.2)	83 (64.8)	
M status				0.034*
negative (M0)	127	29 (93.5)	98 (76.6)	
positive (M1)	32	2 (6.5)	30 (23.4)	

^a^Chi-square test

*P < 0.05

### Cell culture

Three human lung adenocarcinoma cancer cell lines (A549, PC-9 and GLC-82), a human squamous lung cancer cell line (L78), and three human bronchial epithelial cell lines (16HBE, HBE-PIC and BEP-2D), one human normal pulmonary epithelial cell line (BEAS-2B) and human embryonic kidney (HEK) 293 T cell lines were purchased from the Cell Bank of Type Culture Collection of the Chinese Academy of Science, Shanghai Institute of Cell Biology. All cells were grown in RPMI-1640(DMEM) medium (Gibco, life technologies, California, USA) supplemented with 10% fetal bovine serum and penicillin (100UI/ mL)/streptomycin (100 mg/mL) (Gibco, life technologies, California, USA), and were maintained in an incubator at 37 °C with 5% CO_2_.

### Real-time quantitative reverse transcription PCR (RT-qPCR)

Total RNA from tissue and cell lines were extracted using TRIzol reagent (Invitrogen, CA). The concentration of isolated total RNA was measured by NanoDrop ND-1000 Spectrophotometer (Agilent, CA). The total RNA was reversely transcribed by using Super Script III First-Strand Synthesis System for RT-PCR (Invitrogen, CA).

Primers were designed in Primer Express 3.0 and listed in Additional file 1: Table S1. PCR reactions were carried out on an ABI PRISM 7900 HT system using the TaqMan Universal PCR Master Mix (Applied Biosystems). The real-time PCR reactions were performed in triplicate. The relative levels of gene expression were represented as ΔCt = Ct_gene_ − Ct_reference_, and the fold change of gene expression was calculated by the 2^−ΔΔCt^ method.

### Plasmid construction, lentiviral production, and transduction

The pEZ-Lv201-based lentivirus was prepared according to the User Manual of the Lenti-Pac™ HIV Expression Packaging Kit (GeneCopoeia, Inc.). After confirmation of the constructed plasmids by DNA sequencing, the viral packaging was performed in 293Ta cells, or empty lentiviral vector as negative control (pEZ-Lv201-NC, pNC), and Lenti-Pac™ HIV packaging mix (GeneCopoeia, Inc., CatNo. HPK-LvTR-20) using EndoFectin™ Lenti transfection reagent (GeneCopoeia, Inc., CatNo. HPK-LvTR-20). The full-length human lnc-IGFBP4–1 cDNA and small hairpin RNA (shRNA) are both synthesized by iGeneBio (Guangzhou, China), then the lnc-IGFBP4–1 gene sequence was subcloned into the lentiviral expression vector pEZ-Lv201 (GeneCopoeia, Guangzhou, China) (pEZ-Lv201-lnc-IGFBP4–1, pLnc-IGFBP4–1) for up-regulation; small hairpin RNA (shRNA) of lnc-IGFBP4–1 was cloned into vector psi-LVRH1GH for gene silencing. Lung cancer cells cultured in six-well plate were transfected with the pLnc-IGFBP4–1, empty vector, psi-LVRH1GH-lnc-IGFBP4–1 or sh-NC. The medium containing the retroviral supernatant was harvested 48 h after transfection using qRT-PCR.

### Cell proliferation assay

Cell proliferation assay was performed with Cell Counting Kit-8 (CCK-8, Corning Corporation, USA) abiding by the manufacturer’s protocols. Briefly, 1000 cells were cultured in a 96-well plate. 1000 cells were plated into a 6-well plate and maintained in media containing 10% fetal calf serum. OD450 was measured 2 h after adding CCK-8 using a Synergy 2 microplate reader (BioTek Instruments, US) in the 24 h, 48 h, 72 h, 96 h. This experiment was done in quintuplicate cells.

### Flow cytometry assay

For cell cycle analysis, cells, after transfection for 48 h, were harvested by trypsinization and washed twice with PBS, then were fixed overnight in 1 mL of 70% (*v*/v) ice-cold ethanol at 4 °C. The cells were treated with 10 mg/mL RNase at 37 °C for 30 min in an incubator, and then stained with 1 mg/mL propidium iodide (PI) in PBS at 37 °C for 30 min in an incubator with 5% CO_2_, and analyzed immediately by Flow Cytometry (FACScan; BD Biosciences, Shanghai, China) equipped with CellQuest software (BD Biosciences) according to the manufacturer’s guidelines. Cells were classified as viable, dead, early apoptotic, or apoptotic. The percentage of early apoptotic cells was counted and compared between cells receiving different treatment. Cells for cell cycle analysis were stained with propidium iodide using the BD Cycletest Plus DNA Reagent Kit (BD Biosciences). The relative ratio of cells in G0/G1, S, or G2/M phase was counted and respectively compared with control groups. Each experiment was performed in triplicate.

### Plate clone formation assay

From each group, nearly 3 × 10^2^ cells were added to each well of a 6-well culture plate. Each cell group consisted of three wells. After incubating at 37 °C for 14–21 days, the cells were washed twice with PBS and stained 0.1% crystal violet. The number of colonies containing ≥50 cells was counted under a microscope [plate clone formation efficiency = (number of colonies/number of cells inoculated) ×100%]. These experiments were performed in triplicate.

### Migration and invasion assay

The 24-well BD BioCoat Matrigel Invasion Chambers were used as per the manufacturer guideline (BD Bioscience). 2–4 × 10^5^ cells were added to the upper wells separated by an 8 μm pore size PET membrane with a thin layer of matrigel basement membrane matrix (for invasion) or without (for migration). The membranes were stained with Diff Quick stain (Fisher Scientific) after removing the non-migrated cells from the top of the membrane with Q-tips. After air-drying, the membranes were cut and mounted on slides with oil, and cells that had migrated to the underside of the filter were counted using light microscope (Zeiss Axio Observer) in five randomly selected fields (magnification; 40×). Each assay was performed in triplicate.

### Tumor xenograft

Five-week-old female BALB/C-nu mice, purchased from the Center of Experimental Animals, Guangzhou University of Chinese Medicine, were housed and maintained in laminar airflow cabinets under specific pathogen-free conditions. The stable transfected lung cancer cell sublines were collected and resuspended in PBS. The stable transfected PC-9 cells with overexpression lnc-IGFBP4–1 or control cells were injected subcutaneously into BALB/C-nu mice (1 × 10^7^ cells/mice in 200 μl PBS), respectively. Each tumor cell subline was injected into 6 mice. After injected one week, the mice were examined 3 times per week for 3 weeks, and tumor growth was evaluated by measuring the length and width of tumor mass. All experimental protocols were reviewed and approved by the Ethic Committee on Animal Experimentation of Guangzhou Medical University.

### Quantitative determination of intracellular ATP level

The intracellular ATP level was measured using the CellTiter-Glo Luminescent Cell Viability Assay kit. Cells were cultured at 5 × 10^3^ cells per well in a 96-well plate in RPMI-1640(DMEM) medium supplemented with 10% FBS, and allowed to grow overnight. Cells were given treatment with 2-DG (2.5 mmol/L), Rho123 (1.5 μg/ml) and the mixture of 2-DG (2.5 mmol/l) and Rho123 (1.5 μg/ml) for 6 h, respectively. The ATP level in cells was then determinate using a CellTiter-Glo Luminescent Cell Viability Assay kit according to the manufacturer’s protocol. Luminescence was measured using the Glomax Multi Plus Detection System (Promega, Madison, WI, USA). The luminescence signal is proportional to the amount of ATP as an index of cell number.

### Statistical methods

All statistical data were analyzed by Stata11.0 software (Stata, College Station, TX, USA). Chi-square test and logistic regression analysis were used to analyze the association among the expression level of gene, the clinic features and the progression of lung cancer; paired T test were used to analyze the expression level of gene between lung cancer tissue and normal tumor-adjacent lung tissues; Independent t test were used to analyze the account of clone formation, cell migration and invasion ability, the expression level of Tumor cell sugar metabolism related enzymes; repetitive measurement deviation analysis were used to analyze the difference of cell proliferation and the tumor of cell proliferation. All statistical tests were two-sided, and *P* < 0.05 was considered to be statistically significant.

## Results

### The lnc-IGFBP4–1 is upregulated in LC tissues and correlates with clinicopathological features

**Fig. 1:**
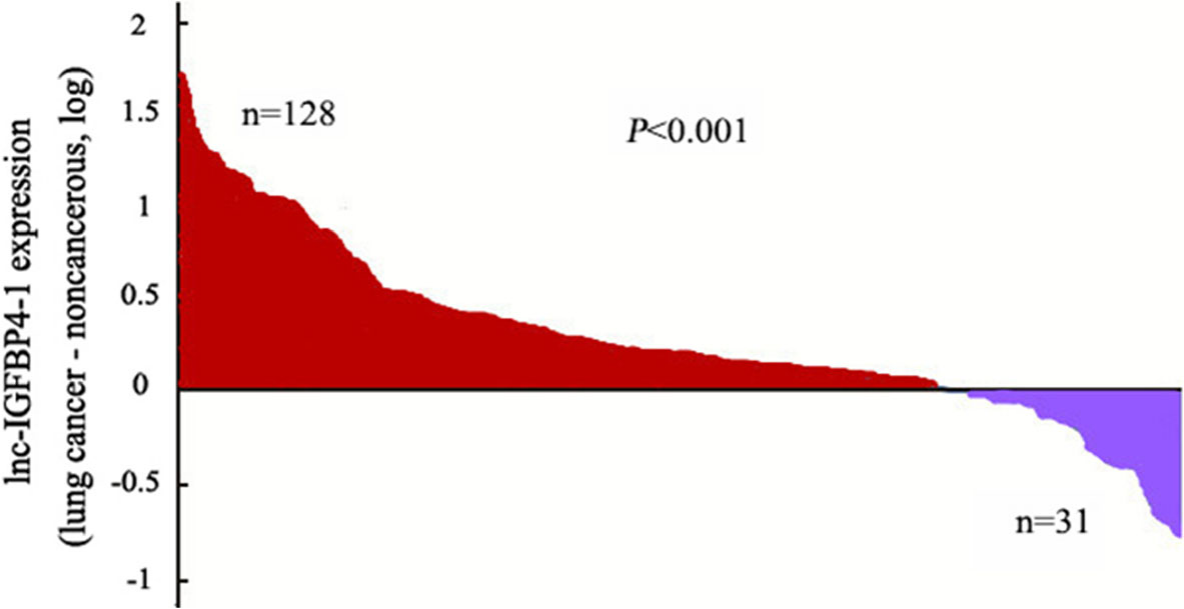
Lnc-IGFBP4–1 expression in human lung cancer (LC) tissues. Relative expression of lnc-IGFBP4–1 in LC tissues (*n* = 159) compared with corresponding non-tumor tissues (n = 159). Red: up-regulation; purple: down-regulation

**Fig. 2:**
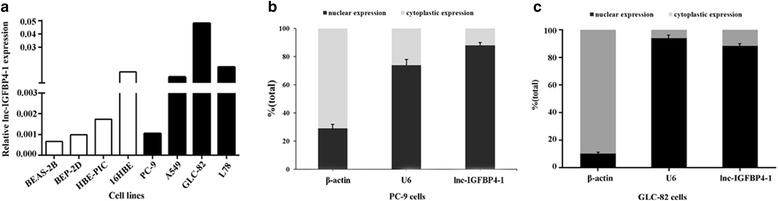
Lnc-IGFBP4–1 expression in LC cells. **a** qRT-PCR results demonstrating lnc-IGFBP4–1expression in four LC cell lines (PC9, A549, GLC-82, and L78) compared to human normal bronchial/pulmonary epithelial cell lines (16HBE, HBE-PIC BEP-2D and BEAS-2B). **b**, **c** lnc-IGFBP4–1 nuclear localization, as identified using qRT-PCR in fractionated PC9 cells and GLC-82 cells. β-actin was used as a cytosol marker and U6 was used as a nucleus marker

To validate whether lnc-IGFBP4–1 was differentially expressed in LC tissues, a total of 159 paired clinical LC tissues and adjacent normal counterparts were examined for lnc-IGFBP4–1 expression by qRT-PCR. Lnc-IGFBP4–1was significantly over-expressed in cancerous tissues (*P* < 0.01; Fig. 1). Additionally, to assess the clinical significance of lnc-IGFBP4–1, we evaluated the correlation of its expression with clinicopathological characteristics, as shown in Table 1, the lnc-IGFBP4–1 level was associated with TNM stage (*P* = 0.007), primary tumor (*P* = 0.041), regional lymph nodes (*P* = 0.044), distance metastasis (*P* = 0.034). However, there was no significant correlation between lnc-IGFBP4–1 expression and other clinicopathological features, such as age, gender, smoking status and drinking status (*P* > 0.05). Additionally, we next conducted qRT-PCR analysis to examined lnc-IGFBP4–1 expression in LC cell lines, involving both adenocarcinoma and squamous carcinoma subtypes. In most cancerous cells, lnc-IGFBP4–1 expression was at a higher level than that of control normal cells. In contrast, the relatively lower expression levels of lnc-IGFBP4–1 were observed in PC9 cell line (Fig. 2a). Subcellular locations maybe provide clues about the molecular mechanism, β-actin and small nuclear RNA U6 (RNU6) were employed as fractionation indicators. Compared with β-actin and RNU6, lnc-IGFBP4–1 was predominantly located in nucleus (Fig. 2b and c), implying that lnc-IGFBP4–1 was mainly localized in the nucleus and maybe function as a major regulator at the transcriptional level. These data implied that the over-expressed lnc-IGFBP4–1 may serve as important regulator in LC development and progression.

### Lnc-IGFBP4–1 promotes cell proliferation and apoptosis in vitro

**Fig. 3:**
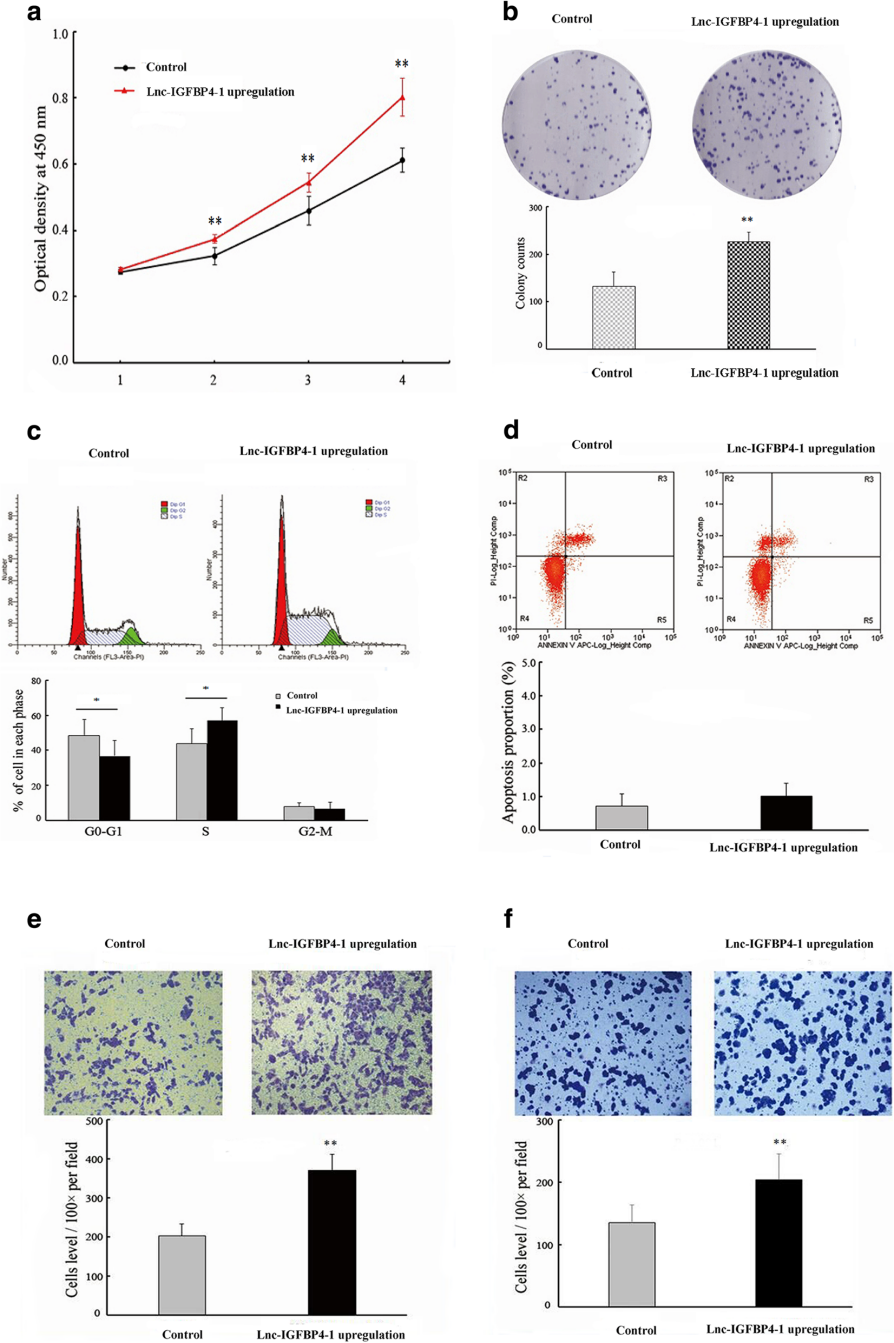
Upregulation of endogenous lnc-IGFBP4–1 promotes lung cancer cell proliferation in vitro. PC9 cells were transfected with lnc-IGFBP4–1-upregulation vector (pLnc-IGFBP4–1) (or empty vector as control), respectively. **a** CCK8 assay and **b** clone formation were performed to determined the cell proliferation. The results from three independent experiments, showed as mean ± s.d. **c** Cell-cycle analysis was conducted by flow cytometric analysis and showed cell division at the G0 – G1 stage. **d** Apoptosis was detected by flow cytometry. UL, necrotic cells; UR, terminal apoptotic cells; LR, early apoptotic cells. **e** and **f** Migration and invasion capacities determined by Transwell assays. *P < 0.05, **P < 0.01

**Fig. 4:**
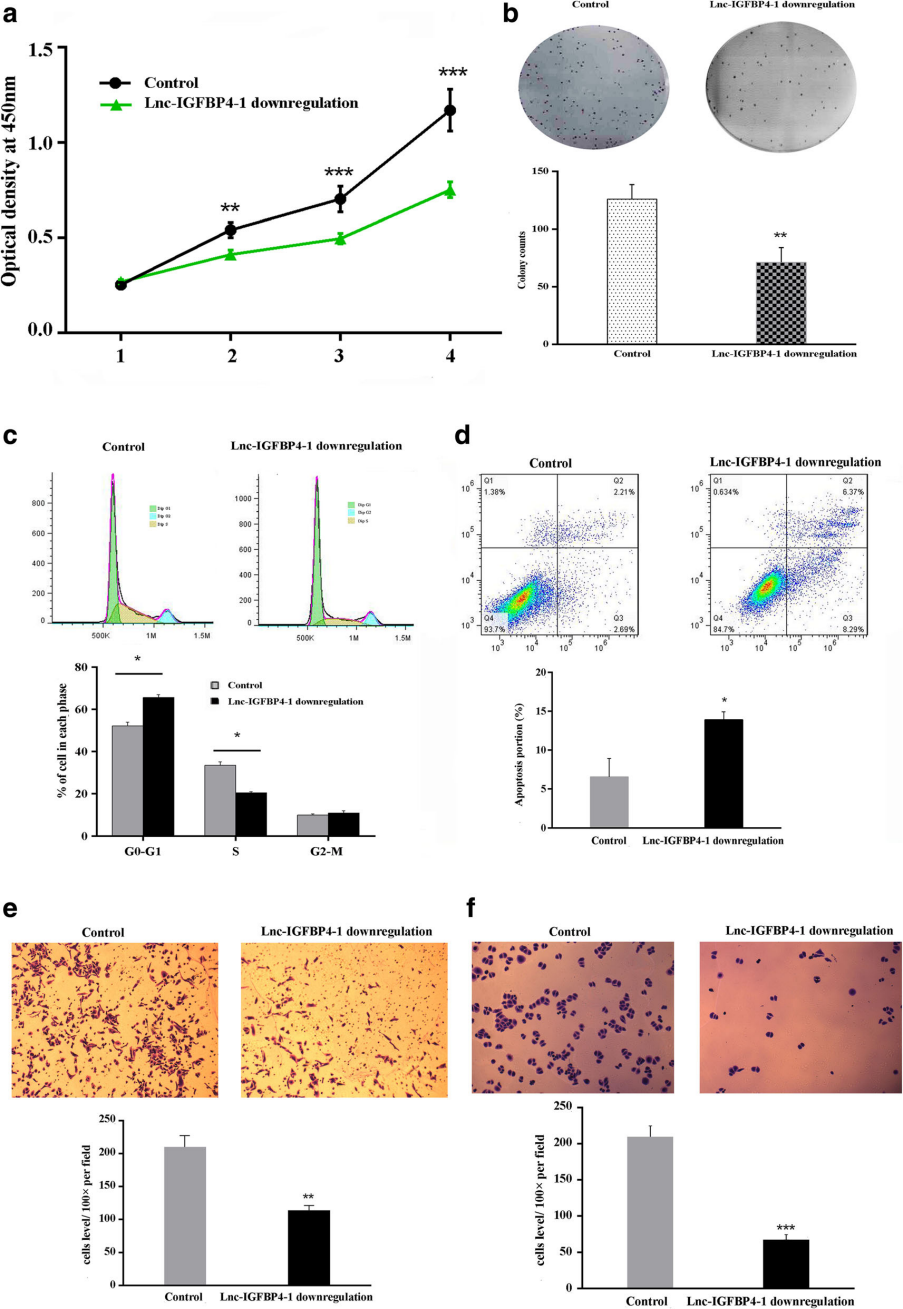
Downregulation of endogenous lnc-IGFBP4–1 inhibits lung cancer cell proliferation in vitro. GLC-82 cells were transfected with lnc-IGFBP4–1-downregulation vector (sh-Lnc-IGFBP4–1) (or empty vector as control), respectively. **a** CCK8 assay and **b** clone formation were conducted to determined the cell proliferation. The results from three independent experiments, showed as mean ± s.d. **c** Cell-cycle analysis was conducted by flow cytometric analysis and showed cell cycle arrest at the G0 – G1 stage. **d** Apoptosis was detected by flow cytometry and promoted apoptosis. UL, necrotic cells; UR, terminal apoptotic cells; LR, early apoptotic cells. **e** and **f** Migration and invasion capacities determined by Transwell assays. *P < 0.05, **P < 0.01

To further explore the effect of lnc-IGFBP4–1 on cell biological behaviors, we investigated the effect of upregulation or downregulation of lnc-IGFBP4–1 on cell proliferation and apoptosis. Compared with the control cells, the significantly increased cell proliferation ability of PC9 transfected with over-expressed lnc-IGFBP4–1 was observed based on the CCK-8 assay (Fig. 3a), while proliferation was decreased in GLC-82 transfected with down-expressed lnc-IGFBP4–1compared with control cells (Fig. 4a). Similarly, colony formation assay results revealed that clonogenic survival was increased following transfection with upregulated lnc-IGFBP4–1 in PC9 cells (Fig. 3b), while declined in sh-lnc-IGFBP4–1 transfected GLC-82 cells (Fig. 4b).We next examined whether the tumor cell cycle was affected after lnc-IGFBP4–1 upregulatiom or lnc-IGFBP4–1 knock-down by Flow cytometry assay. The results revealed that lnc-IGFBP4–1-upregulation treatment decrease the percentage of cells in the G0 – G1 phase, and increase the percentage of cells in the S phase, which may promote cell division (Fig. 3c), while shRNA-mediated knockdown of lnc-GFBP4–1 in GLC-82cells promote G1 arrest. Additionally, flow cytometry analysis of PC-9 and GLC-82 cells showed that downregulation of lnc-IGFBP4–1 expression in GLC-82cells induced apoptosis in comparison with the control cells (Figs. 3d and 4d). Moreover, transwell assays were conducted to determine whether the upregulation or downregulation of lnc-IGFBP4–1 expression can influence LC migration and invasion. Increased lnc-IGFBP4–1 expression promoted PC9 cell migration by 81%, when transfected with lnc-IGFBP4–1-upregulation (Fig. 3e), similarly, PC9 cell invasion was also increased by 51% (Fig. 3f). While down-regulation of inc-IGFBP4–1 expression decreased GCL-82 cell migration and invasion by 45% and 56% by shRNA (Fig. 4e and f). These findings support the conclusion that lnc-IGFBP4–1 exerts an important effect on the promotion of LC cell migration and invasion.

### The upregulated lnc-IGFBP4–1 promotes tumor growth in vivo

**Fig. 5:**
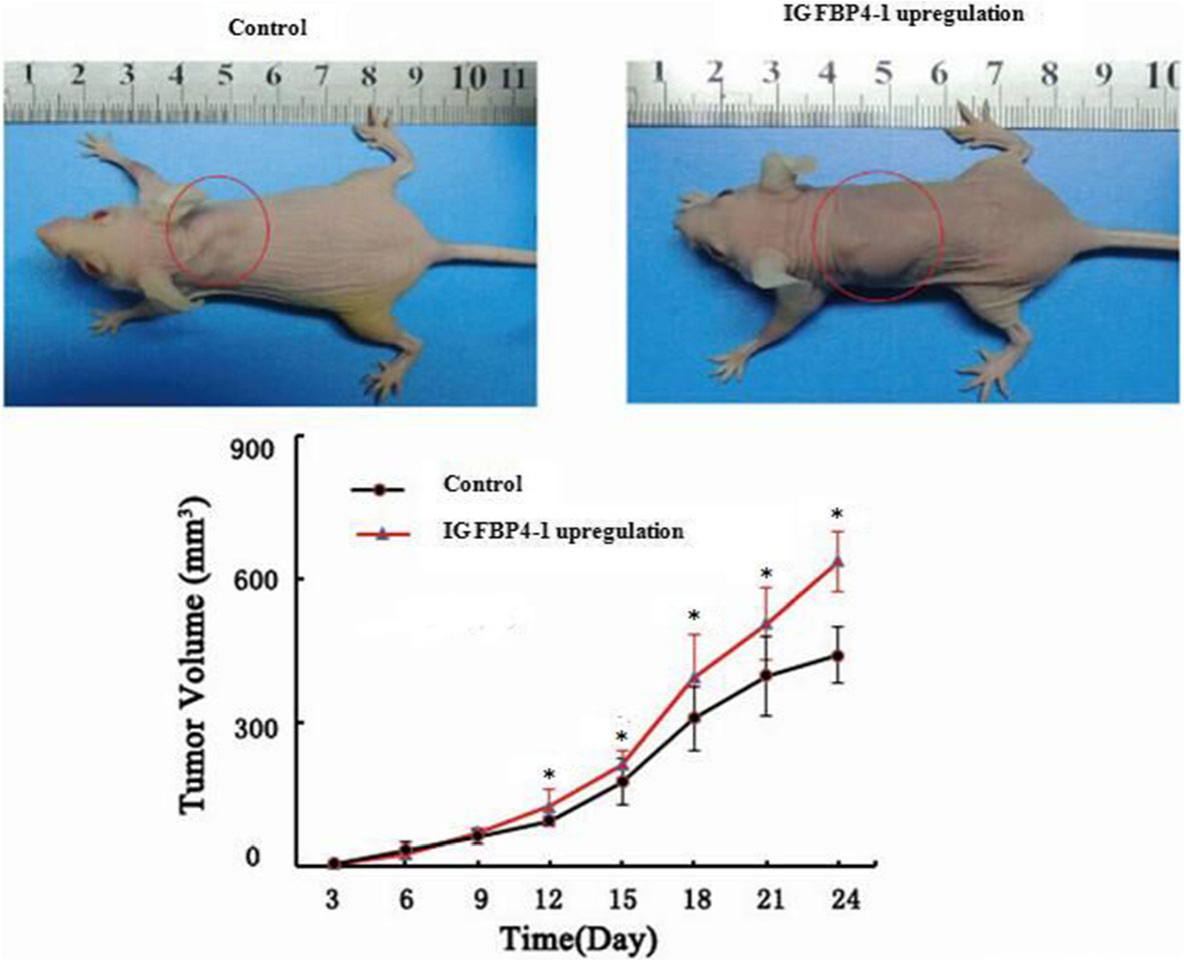
Ovexpressing-lnc-IGFBP4–1promotes PC9 cells proliferation in vivo. PC9 cells transfected with lnc-IGFBP4–1-overexpression were subcutaneously inoculated into nude mice and tumor weight in lnc-IGFBP4-1-overexpression lentivirus-treated group was significantly more than in the control group﻿.﻿^*^ *P* ﻿< 0.05﻿. *n* = 6 mice per group

To evaluate the effect of lnc-IGFBP4–1 on lung carcinogenesis in vivo, we established xenograft tumor models in nude mice with PC-9 cells transfected with overexpression-lnc-IGFBP4–1 treatment. Xenograft tumors were observed in nude mice at the injection site, and the xenograft tumors were harvested 24 days after injection. As shown in Fig. 5, the tumor volume in the overexpressed lnc-IGFBP4–1treatment group was significantly increase compared to that in the control group, and tumor volume was 636 ± 64.1 mm^3^, which was significantly bigger than that in control group (441 ± 58.5 mm^3^) on the day 24 (*P* < 0.05). These results provided further evidence that lnc-IGFBP4–1 plays a tumor promotive role in LC.

### Lnc-IGFBP4–1 regulates energy metabolism of lung cancer.

**Fig. 6:**
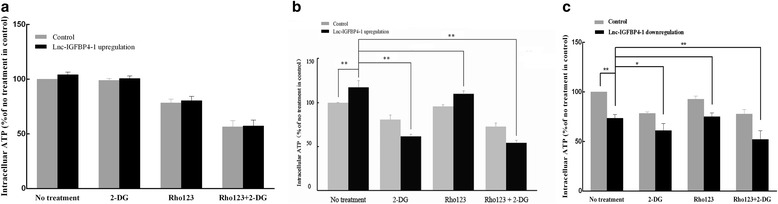
Effects of lnc-IGFBP4–1 on ATP levels. Bar chart exhibited the differences in ATP levels in (**a**) lnc-IGFBP4–1-overexpressing-BEAS-2B cells (control cells), in (**b**) lnc-IGFBP4–1-overexpressing-PC9 cells, and in (**c**) lnc-IGFBP4–1-downexpressing GCLC-829 cells after addition of 2-DG, Rho123, or 2DG + Rho123. The ATP levels in different cells without any treatment were used as baseline to compare with other treatment. Student’s t-test; *P < 0.05, **P < 0.01

Given that tumor cells often develop metabolism alteration to manage the demand of cell-mass increase during cell growth, we then explored whether the proliferation-associated lnc-IGFBP4–1 is complicated in metabolic reprogramming. As showed in Fig. 6a, BEAS-2B cells transfected with lnc-IGFBP4–1 upregulation did not promote energy metabolism compared with control cells following treatment with 2-deoxy-D-glucose (2-DG, an inhibitor of glycolysis), rhodamine 123 (Rho123, an inhibitor of mitochondrial oxidative phosphorylation) and 2-DG-combined Rho123, respectively. We then found that ATP levels in lnc-IGFBP4–1-overexpressing cells increased by 17.5% compared to control cells (*P* < 0.001), and ATP levels were analyzed after the addition of 2-DG Rho123 and 2-DG-combined Rho123, respectively. Compared to that in lnc-IGFBP4–1-overexpressing cells without any treatment, we found ATP levels decreased 49.5% in response to 2-DG, and decreased 53.8% in response to 2-DG-combined Rho123 (all *P* < 0.001) (Fig. 6b). While ATP levels in lnc-IGFBP4–1-downexpressing cells decreased by 19.3% compared to control cells (*P* < 0.001), and ATP levels were analyzed following same treatment. Compared to that in lnc-IGFBP4–1-downexpressing cells without any treatment, we found ATP levels decreased 14.5% in response to 2-DG (*P* < 0.05), and decreased 23.6% in response to 2-DG-combined Rho123 (*P* < 0.01) (Fig. 6c), indicating elevated aerobic glycolysis by lnc-IGFBP4–1 in regulation the intracellular ATP.

### Lnc-IGFBP4–1 regulates metabolic proteins

**Fig. 7:**
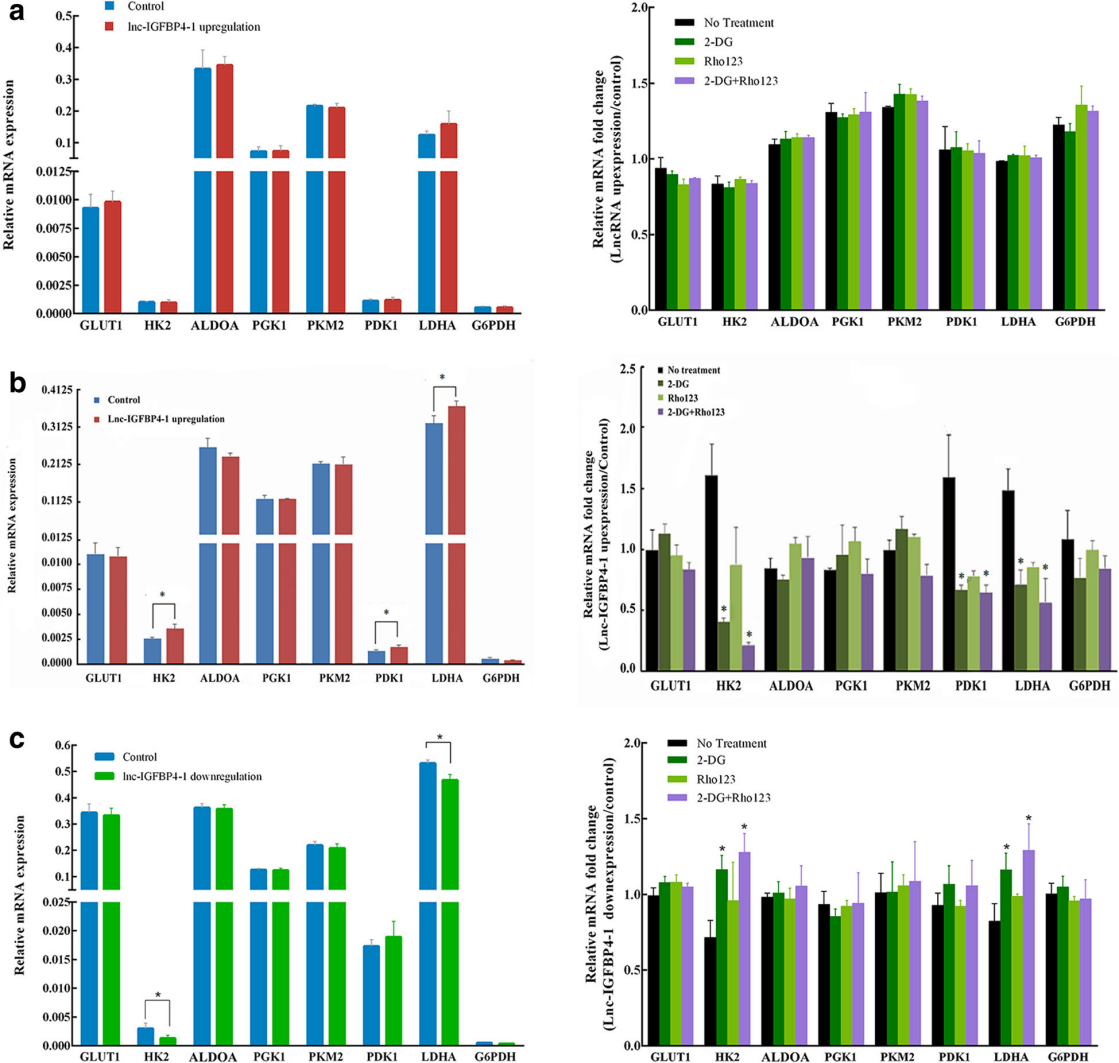
lnc-IGFBP4–1 regulates expression of metabolic enzymes. Expression of the metabolic genes in (**a**) lnc-IGFBP4–1-overexpressing BEAS-2B cells, in (**b**) lnc-IGFBP4–1-overexpressing PC9 cells and in (**c**) lnc-IGFBP4–1-downexpressing GLC-82 cells were determined compared to control cells, and difference in relative metabolic genes fold change after addition of 2-DG, Rho123, or 2DG + Rho123 compared to control cells with no treatment was examined. *P < 0.05, **P < 0.01

To explore how lnc-IGFBP4–1 regulated cellular metabolism, we examined expression of metabolic enzymes in lnc-IGFBP4–1-overexpressing cells or lnc-IGFBP4–1-downexpressing cells, and found that the lnc-IGFBP4–1-induced metabolic alterations take place at the transcriptional level. We determined several enzymes including glucose transporter (GLUT1), human kallikrein 2 (HK2), Aldolase A (ALDOA), phosphoglycerate kinase (PGK1), pyruvate kinase M2 (PKM2), phosphoinositide-dependent kinase (PDK1), lactate dehydrogenase A (LDHA), and glucose-6-phosphatedehydrogenase (G6PDH), implicated in glucose uptake and glycolysis, no difference was observed in enzymes levels in BEAS-2B cells transfected with lnc-IGFBP4–1-upregulation compared with control cells (Fig. 7a); of these enzymes, the expression levels of HK2, PDK1 and LDHA in lnc-IGFBP4–1-overexpressing cells were significantly enhanced than those in control cells (all *P* < 0.05) (Fig. 7b), while expression levels of HK2 and LDHA in lnc-IGFBP4–1-downexpressing cells were inhibited compared with control cells (all *P* < 0.05)) (Fig. 7c). Besides, lnc-IGFBP4-overexpressing cells or lnc-IGFBP4-downexpressing cells were treated with 2-DG, Rho123, and 2-DG combined Rh123, respectively. As shown in Fig. 7b, enzymes expression in lnc-IGFBP4–1-overexpressing cells were more sensitive to glycolysis inhibition by 2-DG and 2-DG-combined Rho123, compared to that in control cells with corresponding treatment. These results implied that lnc-IGFBP4–1 functions as an important regulator involved in multiple metabolic activities, whose expression alterations in turn result in metabolic outcomes in favor of tumor cell growth.

### Association of lnc-IGFBP4–1 expression with IGFBP4 expression.

**Fig. 8:**
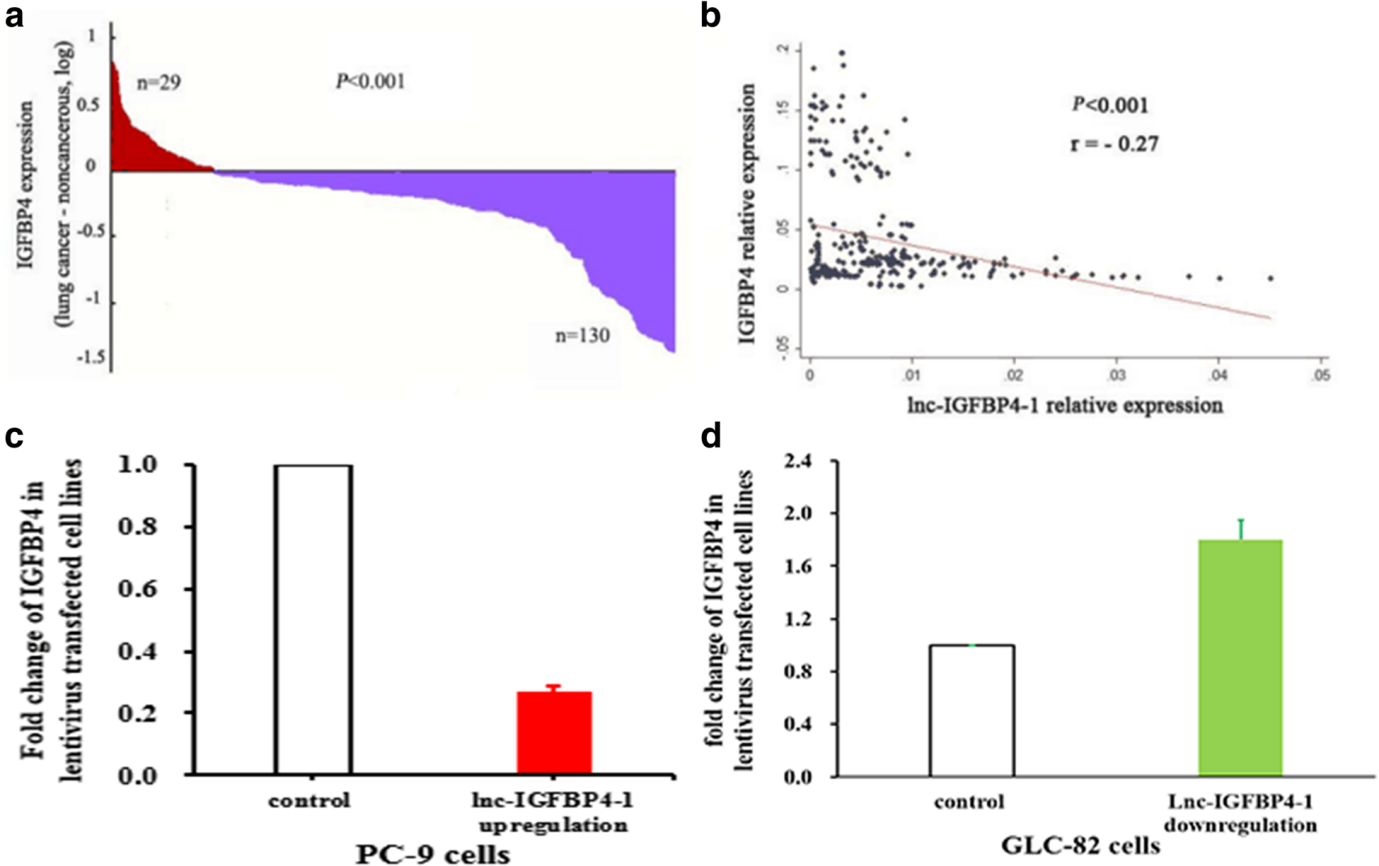
Correlation of lnc-IGFBP4–1 expression with mRNA level of IGFBP4 in LC patients. **a** Relative expression of IGFBP4 in LC tissues (n = 159) compared with corresponding non-tumor tissues (n = 159). Red: up-regulation; purple: down-regulation. **b** The lnc-IGFBP4–1 and IGFBP4 expression levels were negatively correlated in 159 pairs of cancer tissue. **c** IGFBP4 expression levels were downregulated in lnc-IGFBP4–1-overexpressing PC9 cells. **d** IGFBP4 expression levels were upregulated in lnc-IGFBP4–1-downexpressing GLC-82 cells

Recent studies have reported IGFBP-4 is found to inhibit tumour progression via sequestering IGFs and cancer inhibitory effects of IGFBP-4 are generally accepted [14, 26]. We further investigated the functional relevance of the interaction between lnc-IGFBP4–1 and IGFBP4. RT-qPCR performed was to exam the expression of IGFBP4 expression in 159 LC tissues compared with adjacent non-tumor tissues. The results showed that IGFBP4 was significantly down-regulated in LC tissues compared with paired adjacent normal lung tissues *P* < 0.001) (Fig. 8a), and a negative correlation relationship was found between the expression of IGFBP4 and lnc-IGFBP4–1 (*r* = −0.27, *P* < 0.001) (Fig. 8b). Moreover, down-regulated IGFBP4 was observed in lnc-IGFBP4–1-overexpressing cells, and up-regulated IGFBP4 was found in lnc-IGFBP4–1-downexpressing cells (Fig. 8c and d), indicating that lnc-IGFBP4–1 may exert a key role in regulating the gene IGFBP4.

## Discussion

Reprogrammed energy metabolism as an emerging hallmark of cancer acquires metabolic changes in order to sustain rapid proliferation and cell growth and adapt to the tumor microenvironment [22, 27–29]. Recently, increasing evidence has revealed that aberrant expression of lncRNAs may be involved in epigenetic changes and participate in proliferation cancer cell growth or metastasis [30–34]. The latest reports indicate some lncRNAs, such as PCGEMI, UCA1, lincRNA-p21 and CRNDE play a positive role in cancer cell glucose metabolism to contribute to the Warburg effect [10, 35–37], however, it remains largely unknown whether and how lncRNA regulates cellular energy metabolism in lung cancer.

IGFBP-4 is an important member of the IGFBP family of proteins and is expressed in some cancers, such as lung and prostate cancer cells [38–40]. Recently, in vivo studies have showed that IGFBP-4 exerts its anti-proliferative action and inhibits the growth of some cancers [26, 41]. In this study, we conducted bioinformatics analysis and found IGFBP4–1 as a new candidate lncRNA located in the upstream region of IGFBP4 gene. It remains unclear whether lnc-IGFBP4–1has the biological functions and related molecular mechanisms of in LC. Here, we found that the expression of IGFBP-4 was decreased in cancer tissue and a negative correlation relationship between the expression of IGFBP4 and lnc-IGFBP4–1. Additionally, down-regulated IGFBP4 was found when the PC9 cell line was treated with lnc-IGFBP4–1-overexpressing, implying lnc-IGFBP4–1 may exert an important role in regulating the target gene IGFBP4.

Our study has identified the lncRNA IGFBP4–1 served as a key regulator not only in proliferation and metastasis but also in energy metabolic changes in cancer cells. First, we found that the average level of lnc-IGFBP4–1 in lung cancer tissues was significantly higher than those in corresponding non-tumor tissues and lnc-IGFBP4–1 expression was significantly correlated with TNM stage and lymph node metastasis, indicative of lncRNA-IGFBP4–1 as a key regulator in LC progression and as a potential novel biomarker for LC. To further explore the biological functions of lncRNA-IGFBP4–1 in LC, we conducted a series of assays both in vitro and in vivo. Further investigation showed that overexpression of lnc-IGFBP4–1 significantly promoted LC cell proliferation in vitro and in vivo, while downregulation of endogenous lnc-IGFBP4–1 could inhibited cell proliferation and induce apoptosis. Tumor cell cycle showed overexpression of lncRNA-IGFBP4–1treatment reduce the percentage of cells in the G0 – G1 phase, and increase the percentage of cells in the S phase, which possibly promote cell division. While shRNA-mediated knockdown of lnc-GFBP4–1 in GLC-82cells promote G1 arrest. Additionally, flow cytometry analysis of PC-9 and GLC-82 cells showed that downregulation of lnc-IGFBP4–1 expression in GLC-82cells induced apoptosis in comparison with the control cells. Furthermore, the lnc-IGFBP4–1 played a positive role in cancerous cells migration and invasion. Taken together, these findings suggest that lnc-IGFBP4–1 could function as a tumor promoter via regulating cell growth and inhibit apoptosis, and could be as a new biomarker for LC. In addition, xenograft tumor models showed overexpression of lnc-IGFBP4–1treatment resulted in bigger tumor volume. These results suggest that lnc-IGFBP4–1 may exert a tumor stimulator-like function in LC.

As we known, cancer cells reprogram energy metabolism to facilitate cell growth and proliferation, the phenomenon is regarded as an emerging hallmark of cancer. Distinct from normal cells, cancer cells preferentially use aerobic glycolysis to metabolize glucose as a result of demanding high energy production and generating low ATP due to the Warburg effect [22] . To compensate for the consequent ATP reduction and biosynthetic intermediates, most cancer cells often adopt corresponding mechanisms to enhance glucose uptake and utilization. In this report, we identified the lnc-IGFBP4–1as an important regulator of energy metabolism that probably provide proliferating advantages for LC cells. We observed that lnc-IGFBP4-overexpressing cells showed significantly increased ATP production, while under the treatment with 2-DG and 2-DG-combined Rho123, ATP production was significantly decreased, indicative of an enhanced aerobic glycolysis rate. Additionally, overexpression of lnc-IGFBP4 facilitates enzymes metabolism, after treatment with 2-DG and 2-DG-combined Rho123, we found a significant decrease level of HK2, LDHA and PDK1. These findings suggest an essential role of lnc-IGFBP4–1in tumor metabolic regulation which possibly provides growth advantages for cancer-cell.

In summary, as mentioned above, lnc-IGFBP4–1 may be a promoter of cancerous cell growth thus linking reprogrammed cellular energy metabolism with lung carcinogenesis. There are several limitations that should be noted. First, due to the limited follow-up information, the prognostic performance of lnc-IGFBP4–1 has not been validated. Second, at present, we know little about the detailed mechanism by which how lnc-IGFBP4–1 regulate or reprogram the cellular energy metabolism to promote cancerous cells proliferate, invade and metastasize, our future work will focus on the mechanisms of how lnc-IGFBP4–1 serves as a tumor promoter gene involved in LC development by regulating metabolic programming. However, regarding the roles of lncRNAs in reprogramming metabolism are anticipated but have not been extensively investigated, to our knowledge, our present study provides an important clue for future investigation of lnc-IGFBP4–1 as a biomarkers for LC and the comprehensive understanding of the energy metabolism-based molecular mechanisms by which lnc-IGFBP4–1 affects LC.

## Conclusions

In conclusion, our results provide the first evidence that lnc-IGFBP4–1 as a novel potential promoter to facilitate cell proliferation, invasion and migration ability highly possibly through cellular energy metabolism process. We found that overexpression of lnc-IGFBP4–1 in cancerous cells notably promoted tumor growth and metastasis both in vivo and in vitro, while downregulation of endogenous lnc-IGFBP4–1 could inhibited cell proliferation and induce apoptosis. In addition, increase in ATP production and metabolic enzymes including HK2, PDK1 and LDHA, respectively, were observed in lnc-IGFBP4–1-overexpressing cells, raising the possibility that lnc-IGFBP4–1 may be a promising new biomarker and therapeutic target for LC.

## Additional file

Additional file 1: Table S1. Sequence of primers using for qRT-PCR analysis (DOCX 12 kb)

## Abbreviations

ALDOA: Aldolase A
G6PDH: glucose-6-phosphatedehydrogenase
GLUT1: glucose transporter
HK2: human kallikrein 2
IGFBP4–1: insulin-like growth factor binding protein 4–1
LC: lung cancer
LDHA: lactate dehydrogenase A
lncRNA: long non-coding RNA
PDK1: phosphoinositide-dependent kinase
PGK1: phosphoglycerate kinase
PKM2: pyruvate kinase M2
