# Supplementary material for: Overexpression of lncRNA IGFBP4–1 reprograms energy metabolism to promote lung cancer progression
Source: Mol Cancer. 2017 Sep 25;16:154. doi: 10.1186/s12943-017-0722-8 (PMC5613386; doi:10.1186/s12943-017-0722-8)
Supplement: Additional file 1: Table S1. — Sequence of primers using for qRT-PCR analysis (DOCX 12 kb) [file 12943_2017_722_MOESM1_ESM.docx]

| Table S1. Sequence of primers using for qRT-PCR analysis | |
| --- | --- |
| **Primer name** | **5**′**-3**′ |
| lnc-IGFBP4-1-F | 5′-GGTGGGGATGTTTGATTT-3′ |
| lnc-IGFBP4-1-R | 5′-CCTGTTTTATGGGCTGAT-3′ |
| IGFBP4-F | 5′-CAGCCCTCTGACAAGGACG-3′ |
| IGFBP4-R | 5′-TCTCGAATTTTGGCGAAGTGC-3′ |
| GLUT1-F | 5′-CCGCAACGAGGAGAACCG-3′ |
| GLUT1-R | 5′-GTGACCTTCTTCTCCCGCATC-3′ |
| HK2-F | 5′-GAATGGGAAGTGGGGTGGAG-3′ |
| HK2-R | 5′-GAGGAGGATGCTCTCGTCCA-3′ |
| ALODA-F | 5′-CAGGGACAAATGGCGAGACT-3′ |
| ALODA-R | 5′-GGATCTCAGGCTCCACGATG-3′ |
| PGK1-F | 5′-CCACTGTGGCTTCTGGCATA-3′ |
| PGK1-R | 5′-ATGAGAGCTTTGGTTCCCCG-3′ |
| PKM2-F | 5′-ACGAGAACATCCTGTGGCTG-3′ |
| PKM2-R | 5′-AGGAAGTCGGCACCTTTCTG-3′ |
| PDK1-F | 5′-GGTGTTTACCCCCCTATTCAAG-3′ |
| PDK1-R | 5′-CGGGAGGTCTCAACACGA-3′ |
| LDHA-F | 5′-AGCCCGATTCCGTTACCT-3′ |
| LDHA-R | 5′-CACCAGCAACATTCATTCCA-3′ |
| G6PDH-F | 5′-TGGAGATCATCATGAAAGAGACC-3′ |
| G6PDH-R | 5′-GCGAATGACACCGTACTCCT-3′ |
| β-actin-F | 5′- GGCGGCACCACCATGTACCCT -3′ |
| β-actin-R | 5′- AGG GGCCGGACTCGTCATACT -3′ |
